# Recognizing Physical Activities for Spinal Cord Injury Rehabilitation Using Wearable Sensors

**DOI:** 10.3390/s21165479

**Published:** 2021-08-14

**Authors:** Nora Alhammad, Hmood Al-Dossari

**Affiliations:** College of Computer and Information Sciences, King Saud University, Riyadh 11584, Saudi Arabia; hzaldossari@ksu.edu.sa

**Keywords:** activity recognition, machine learning, wearable sensors, accelerometers, spinal cord injury, telerehabilitation

## Abstract

The research area of activity recognition is fast growing with diverse applications. However, advances in this field have not yet been used to monitor the rehabilitation of individuals with spinal cord injury. Noteworthily, relying on patient surveys to assess adherence can undermine the outcomes of rehabilitation. Therefore, this paper presents and implements a systematic activity recognition method to recognize physical activities applied by subjects during rehabilitation for spinal cord injury. In the method, raw sensor data are divided into fragments using a dynamic segmentation technique, providing higher recognition performance compared to the sliding window, which is a commonly used approach. To develop the method and build a predictive model, a machine learning approach was adopted. The proposed method was evaluated on a dataset obtained from a single wrist-worn accelerometer. The results demonstrated the effectiveness of the proposed method in recognizing all of the activities that were examined, and it achieved an overall accuracy of 96.86%.

## 1. Introduction

Rehabilitation is necessary for patients who suffer from inhibited mobility due to conditions such as spinal cord injuries (SCI). Rehabilitative therapies and activities can lead to the development of motor skills in patients, which are essential for daily life activities [1]. Rehabilitation also helps to avoid associated symptoms such as obesity and low muscular strength, which can lead to cardiovascular diseases, diabetes, and other complications [2,3]. Different approaches are used to rehabilitate SCI patients, including in-home physical therapy [4]. Currently, physiotherapists rely only on patient surveys and self-reported measures to verify patient adherence to prescribed physical activity. However, several studies have demonstrated that self-reported measures cannot adequately evaluate physical activities that have been performed, which may severely affect the progress of rehabilitation [5]. Therefore, more reliable methods should be adopted to detect these activities.

**Figure 1:**
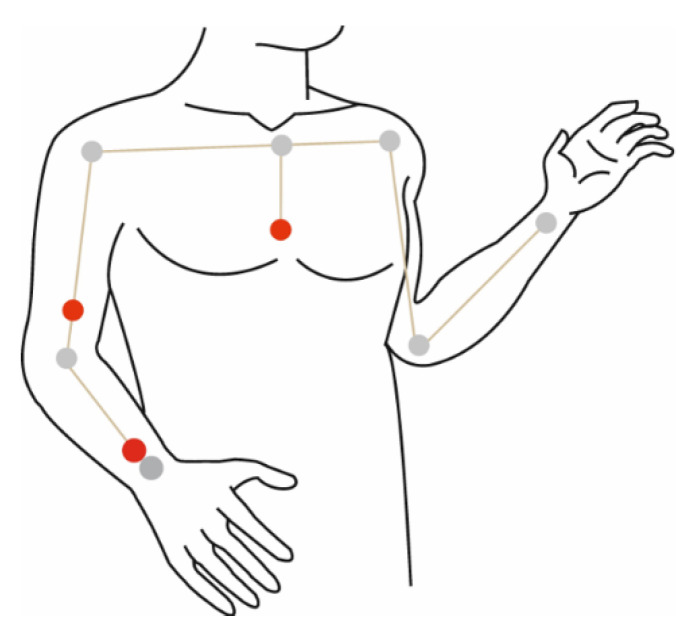
Biomechanical model of the human body.

Given today’s rapid innovations in information technology, paired with recent advances in wearable sensors, the field of activity recognition has gained more attention, especially in healthcare applications. These applications include activity recognition systems, can promote quality of life for patients, and support independent living without invading privacy. The physical activity recognition systems found in the literature can be categorized into those that follow the biomechanical approach and those based on the machine learning approach. As the name suggests, systems based on the biomechanical approach are developed according to biomechanical models of the human body. These models consist of segments of variable lengths and joints that connect the segments (represented by grey circles in Figure 1). Wearable sensors, mainly inertial measurement units (IMU), are used to capture human motion. To recognize an activity, the resulting joint angle is measured by placing IMUs on related body parts such as the bones, ligaments, and between the muscles (denoted by red circles in Figure 1).

The measurements obtained from IMUs cannot be interpreted as an activity unless calibration is performed [6]. Using this approach, Algohari et al. [7] designed a model to control a robotic arm in order to estimate human joint angles using IMUs. Moreover, the study in [6] estimated different joint angles, including wrist, elbow, and shoulder. Peppoloni et al. [8] proposed a wearable system to assess the muscular efforts and postures of the human upper limbs. Specifically, by measuring joint angles, the system estimated different activities performed by workers while they were undertaking their everyday tasks. In [9], Alvarez et al. proposed a method to reduce work-related injuries by initially identifying the movements performed at workplaces. After calculating joint angles related to upper limb activities, the authors in [10] developed a system to measure the estimation error of joint angles obtained from IMUs. Significantly, the calibration procedure is regarded as one of the main limitations of the biomechanical approach. Since the procedure depends on anatomy, as well as the relationship between anatomical landmarks (which differ from person to person), it must be repeated for each individual. In addition, the procedure must be performed by an expert because any abnormal movement can affect the accuracy of the measurements [11].

In contrast to systems based on the biomechanical approach, models based on the second approach use machine learning algorithms as their classification techniques. The overall process is explained in more detail in Section 2. Using this approach, the study in [12] proposed a system that uses a network of wearable sensors, along with an off-the-shelf smartphone, to identify the intensity of a set of upper-body strength exercises. The proposed approach consisted of a multi-layer hierarchical algorithm that uses support vector machine (SVM) to initially recognize the type of exercise, and then to identify its intensity. The results demonstrated the ability of the algorithm to recognize the type of exercise with approximately 85% in terms of overall accuracy. Biswas et al. [13] developed a methodology to recognize three fundamental upper-limb movements using data collected from a wrist-worn accelerometer and a gyroscope. The methodology drew on different classification algorithms, including linear discriminant analysis (LDA) and SVM, which achieved approximately 88% and 83% in terms of accuracy using data from the accelerometer and gyroscope, respectively. Panwar et al. [14] presented a model with the capability of recognizing three arm movements under real-time conditions. With a wrist-worn tri-axial accelerometer, the average recognition rate reported by the authors was 90% using hybrid evaluation methods.

In the context of rehabilitation monitoring, Lin et al. [15] developed a monitoring system based on wireless sensor networks (WSN) to recognize a set of physical activities that assist in frozen shoulder rehabilitation. Using features extracted from the time and frequency domains, the model achieved an accuracy of 85% to 95% in recognizing these activities. With the aim of rehabilitating stroke patients, Cai et al. [16] developed an upper-limb robotic device to provide mirror therapy to the affected side of a patient by recognizing the motion of their healthy side. The method involved the acquisition of surface electromyography (sEMG) signals for model training and validation, and the classification of activities using SVM algorithm. Since stroke survivors typically experience balance problems, which can affect their rehabilitation, Zambrana et al. [17] proposed an approach to ensure the preservation of correct posture by providing feedback to patients. The approach uses wearable sensors to monitor arm movements, which consists of two sequential levels: the upper level, which is aimed at distinguishing between movements and non-movements; and the lower level, which classifies each movement as either purposeful or not. However, none of the previous studies considered monitoring the rehabilitation of individuals with SCI. Furthermore, based on our previous literature review that examined upper-limb physical activity recognition, a gap appears to exist in terms of recognizing some of the activities needed to rehabilitate SCI patients [18]. To address this issue, this paper proposes a systematic activity recognition method based on machine learning to identify physical activities performed during SCI rehabilitation.

The rest of this paper is organized as follows: Section 2 offers an overview of the proposed method and describes the experimental setup used to verify the method; Section 3 and Section 4 present and discuss the results; and Section 5 concludes the paper.

## 2. Methodology

**Figure 2:**
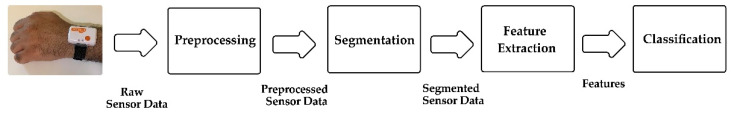
Physical activity recognition pipeline.

To develop the proposed method and build the predictive model, a machine learning approach was adopted. Figure 2 shows the activity recognition pipeline and outlines the structure of the method. The overall process is illustrated in this section. Furthermore, details regarding the experiments used to verify the method are also described in this section.

### 2.1. Rehabilitation Activities

SCI mostly affects the lower limbs, whereas in less common cases, individuals may suffer from complete paralysis based on the degree and location of the injury [19]. This work focuses on the former type of injury, where individuals need rehabilitation to avoid low muscular strength and other associated symptoms, and develop motor skills, which are essential for daily life activities.

**Figure 3:**
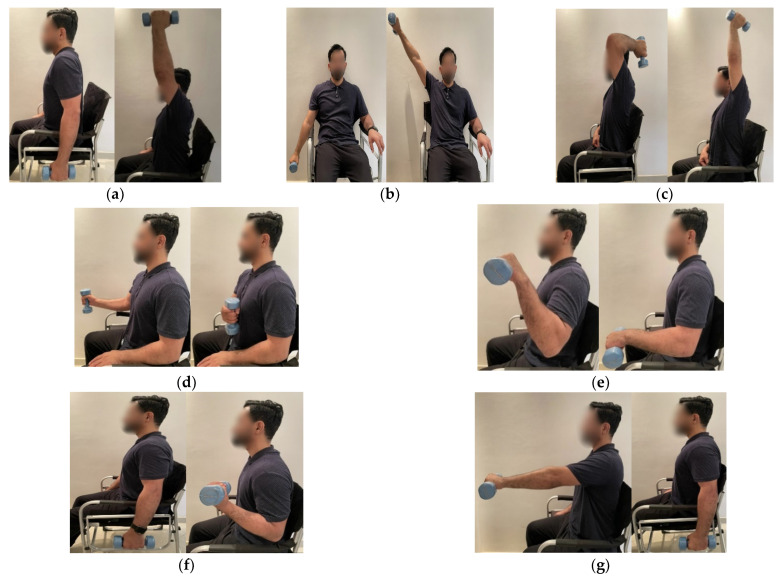
Physical activities used to rehabilitate spinal cord injury (SCI) patients: (**a**) shoulder flexion (SF); (**b**) shoulder abduction (SA); (**c**) elbow extension (EE); (**d**) shoulder internal rotation (SIR); (**e**) shoulder external rotation (SER); (**f**) elbow flexion (EF); and (**g**) shoulder extension (SE).

Where the aim is to strengthen the upper limbs, elbow and shoulder are denoted as the segments of focus [19,20]. Physical activities related to these segments, which have been designed by physiotherapists in [21] to rehabilitate SCI, are shown in Figure 3, along with the execution sequence for each activity.

### 2.2. Instruments and Data Collection

In this study, a single wireless sensor was used (Shimmer Research, Dublin, Ireland). Due to its sufficiently small and lightweight form factor (51 mm × 34 mm × 13 mm in size and 22 g in weight), it can be comfortably worn by patients. It consists of a tri-axial accelerometer with a sensitivity of 660 mV/g, a tri-axial gyroscope, and a tri-axial magnetometer. However, the gyroscope and magnetometer were excluded since studies have demonstrated that accelerometers provide higher overall accuracy compared to gyroscopes, which tend to suffer from high error rates [21,22]. In addition, the ferromagnetic materials available in domestic environments can affect magnetometer measurements. Before use, the device was calibrated according to the manufacturer’s guidelines.

Acceleration data was collected with a sampling frequency of 30 Hz (range ±2 g). This has been proven to be sufficient for the recognition of similar activities [12,23]. The authors in [24] found that even lower sampling rates, including 10 Hz, can be used to recognize the type and intensity of human physical activities.

**Figure 4:**
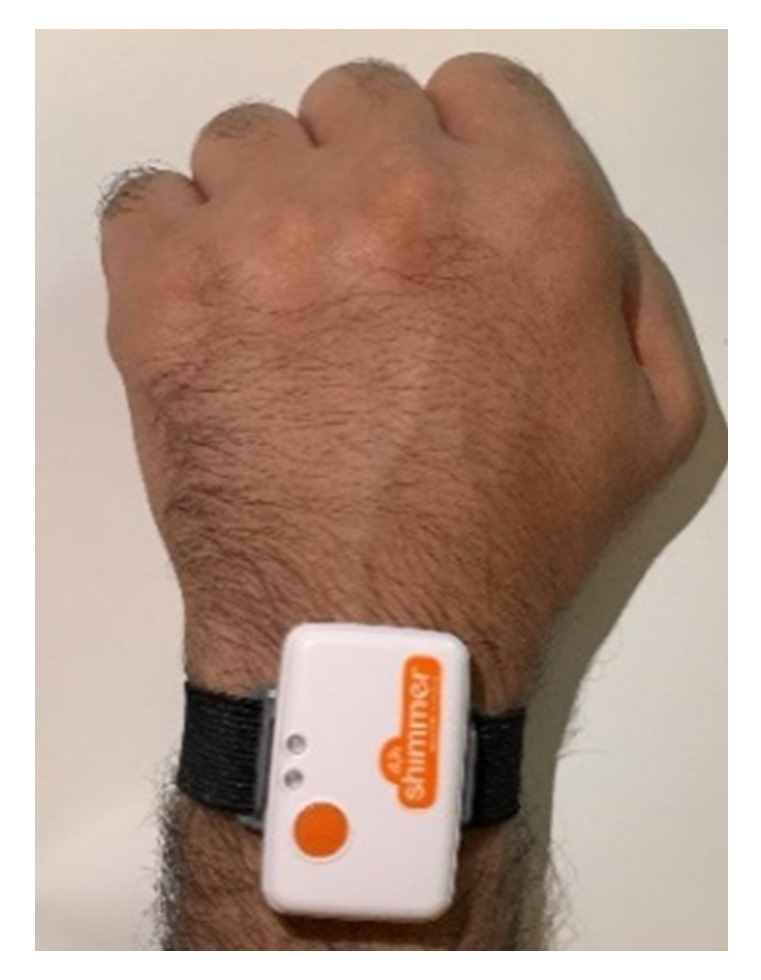
Sensor position.

Substantial studies have been developed around the investigation of the impact of sensor position on overall recognition accuracy. These studies emphasize that sensor position is mainly determined by the activity under study. For upper-limb activities, sensors are placed on the wrist and upper arm to obtain the highest performance [25]. In this research, both positions were tested for data acquisition. However, sensors worn on the upper arm cannot detect certain activities, including EE, EF, and IR, since these activities lack upper arm movements. Consequently, the wrist was selected as the sensing position, as shown in Figure 4. In addition, a meta-analysis was undertaken in [26] to examine user preferences for sensor placement, which is an important aspect for the system to gain the required level of acceptance. The research indicated that people prefer wearing sensors on their wrist, followed by the trunk, belt, and, finally, the ankle.

Ten healthy individuals (3 male, 7 female; aged 29 ± 5.5 years) were asked to perform a series of physical activities. Before the experiment, all participants signed an informed consent form that explained the nature of the procedure. The participants were also given instructions describing the correct execution of the exercises. Finally, a short demonstration video was shown with additional practical advice for optimal performance. For measurements, each participant was asked to perform 10 repetitions of each activity. As a result, acceleration data amounting to 700 repetitions were obtained from the participants.

### 2.3. Data Preprocessing

**Figure 5:**
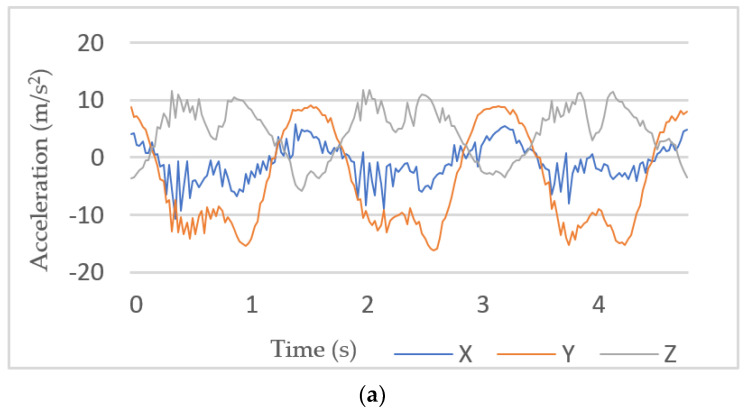

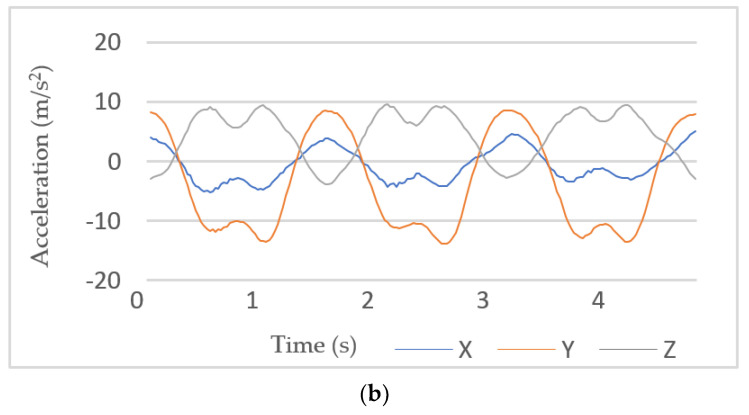
Preprocessing of raw sensor data: (**a**) before preprocessing; and (**b**) after MAF.

Due to the nature of external vibration, and/or sensor errors, the raw data acquired from wearable sensors mostly contain unwanted noise, as shown in Figure 5a. Hence, it is essential to preprocess the raw data to obtain a format that is considered representative of physical activities and suitable for predictive modeling [27,28]. Essentially, all measured body movements are expressed within frequency components below 20 Hz. Thus, it is necessary to remove high-frequency noise without losing information in the lower frequency bands. Different methods are available for noise reduction and smoothing. In this work, moving average filter (MAF) was used (Figure 5b). This has also been selected for similar studies, as in [29,30], which have approved of its efficiency. This is equivalent to low-pass filtering, which makes small perturbations insignificant to the model. Different values were evaluated, and it was found that a length equal to 10 produced smoother data without losing key information.

### 2.4. Segmentation

Before proceeding to feature extraction and the remaining operations, continuous raw data must be divided into smaller fragments. Each fragment corresponds to a specific activity. In this study, the dynamic segmentation method proposed in our previous work [31] was applied. Unlike previous methods, this method is concerned with the segmentation of physical activities belonging to the same category (i.e., dynamic activities). To segment raw data adaptively, the method initially analyzes signal characteristics. Then, it determines the suitable type of boundaries, which can be either peak or valley boundaries, to represent the start and end points of each activity. This dynamic segmentation approach was achieved by following three main steps. The first step involves the selection of peaks in the acceleration signal based on a threshold and a minimum distance, which represent the minimum value of a peak and the minimum distance between peaks in the learning dataset, respectively. The second step is the selection of valleys based on another threshold that represents the highest value of a valley. The final step is the analysis of the signal in each peak to determine the boundaries of each activity. Consequently, the segment’s length will be proportional to the duration of the corresponding activity.

### 2.5. Feature Extraction

To obtain a suitable input for a machine learning algorithm, the data resulting from the previous step are used to calculate multiple aggregate features. These features are used during model training to explore unique patterns, which will be used later for physical activity classification. Different types of features are available, including time- and frequency-domain features, as well as heuristic features. In sensor-based activity recognition, time-domain features have mostly been adopted due to their effectiveness [12,32]. Additionally, they are recommended for use in low-power real-time applications due to their low computation and memory requirements [22]. According to the experiment conducted by [28] to evaluate the performance of three possible feature sets, namely time-domain, frequency-domain and wavelet-domain statistics, and their combinations using data collected from body-worn accelerometer. The highest recognition accuracy was achieved when only time-domain features were used. Furthermore, it has been concluded that time-domain features are most effective in discriminating activities from accelerometer signals.

In this study, different time-domain features, which are commonly used in literature, were extracted from the three axes (*x*, *y*, and *z*) of the acceleration data [12]. Moreover, signal magnitude (*m*) was calculated to extract features ($m=\sqrt{x^{2}+y^{2}+z^{2}}$). A list of the features used, along with their definitions, is shown in Table 1.

### 2.6. Classification

The key step in activity recognition is classification, where the advantage of the extracted features is exploited. Since the outputs of such a problem are controlled, the supervised approach is mostly applied. In addition, the unsupervised approach may result in a more complex model [33]. Accordingly, the former approach was used in this research to reduce computational cost and model complexity.

Several classification algorithms have been proposed in the literature, including SVM, K-nearest neighbor (KNN), decision tree (DT), and Gaussian naïve Bayes (GNB) [34]. In this work, each of these algorithms was examined due to their previous use in similar studies [12,35,36]. In addition, they belong to different classifier types with different internal representations and biases, which leads to different levels of performance. A brief description of these classifiers, along with their configuration details, is given as follows:

- SVM has been proven to be effective in addressing various problems, including activity recognition. SVM’s high accuracy and robustness to noise and overfitting problems have made it popular and one of the leading classifiers in terms of generalization [37,38]. To detect non-linear relations, the radial basis function (RBF), which is one of the most common kernels, was used in this study. Grid search was applied to tune the RBF kernel parameters. As a result, the chosen values for complexity (C) and radius (r) were 2.00 and 0.01, respectively.
- KNN is a simple algorithm that uses the K-closest training observations in the feature space to predict the class of a new entry. It calculates the distance between observations based on Euclidean distance. In this algorithm, the k parameter can be used to control underfitting and overfitting problems. For example, decreasing the value of k can make the model prone to overfitting [17].
- RF is an ensemble classifier, which involves many individual decision trees. To generate a prediction model using RF, it is necessary to define two parameters: first, the number of classification trees; and second, the number of features in each split [39]. In this research, the default values for the parameters were used. This is because several studies have stated that satisfactory results are mostly obtained with these default values [40].
- GNB is one of the main Bayesian classifiers used in literature. Using a naïve method, this classifier determines the probability of an event, which belongs to a certain class, assuming that all the features that are given as input are independent.

### 2.7. Model Training and Validation

K-fold cross-validation and leave-one-out cross-validation (LOOCV) have been commonly used in the literature. For model selection, K-fold is considered to be the most accurate approach, where k = 10 represents the most common value. On the other hand, LOOCV deals with the fact that the data are collected from different subjects. Therefore, it uses data from some subjects for training and data from the remaining for evaluation. In such a way, it reflects inter-subject variability and tests on yet-unseen data. As a result, it is regarded as one of the best approaches for estimating a realistic performance. Furthermore, it can be used to avoid any possible overfitting, which makes it recommended for activity recognition [41,42]. In this work, the model was evaluated using LOOCV, where data from 9 subjects were used for training, after which the evaluation was performed on the remaining subject. This process was repeated until data from each subject were evaluated exactly once, and an average of performance was obtained. To train the model, 10-fold cross-validation was used, which means that data from the 9 subjects were randomly divided into training and test sets with 90% and 10%, respectively [43]. The Waikato Environment for Knowledge Analysis (WEKA) toolkit was used for both training and validation.

## 3. Results

Various performance metrics are available to evaluate classification performance [17]. With regard to similar studies, the commonly used measures were accuracy, which refers to the ratio of correctly predicted observations to the total observations; recall, which refers to the ratio of correctly predicted positive observations to all positive observations in actual class; precision, which refers to the ratio of correctly predicted positive observations to all positive predictions; and *F*1 score, which is a combination of the precision and recall measures. These measures can be expressed as follows:

(1) $$accuracy=\frac{TP+TN}{TP+TN+FP+FN}$$

(2) $$recall=\frac{TP}{TP+FN}$$

(3) $$precision=\frac{TP}{TP+FP}$$

(4) $$F1\;score=2\times \frac{precision\times \;recall}{precision+recall\;}$$

In the above equations, *TP* is true positive, which represents the number of positive observations that were predicted as positive; *FN* is false negative, which measures the number of positive observations that were predicted as negative; *TN* is true-negative, which represents the number of negative observations that were predicted as negative; and *FP* is false positive, which refers to the number of negative observations that were predicted as positive [41].

To evaluate the performance of the proposed method, an experiment was designed in two stages. First, the abovementioned metrics were used to determine the recognition performance while adopting different classification algorithms. In the second stage, to demonstrate the effectiveness of the method using the dynamic segmentation method, the results were compared to the commonly used fixed-size sliding window approach, which is regarded as the best available approach that has been developed to date [36,41,44].

Table 2 summarizes the performance evaluation results for the proposed method derived from the SVM, KNN, RF, and NB classification algorithms. The proposed method achieved an overall accuracy ranging from 94% to 96.86% using the aforementioned classifiers. This indicates an excellent recognition rate and a high level of agreement between the classification results and the true values. Noteworthily, RF outperformed the other classifiers with an accuracy of 96.86%.

**Figure 6:**
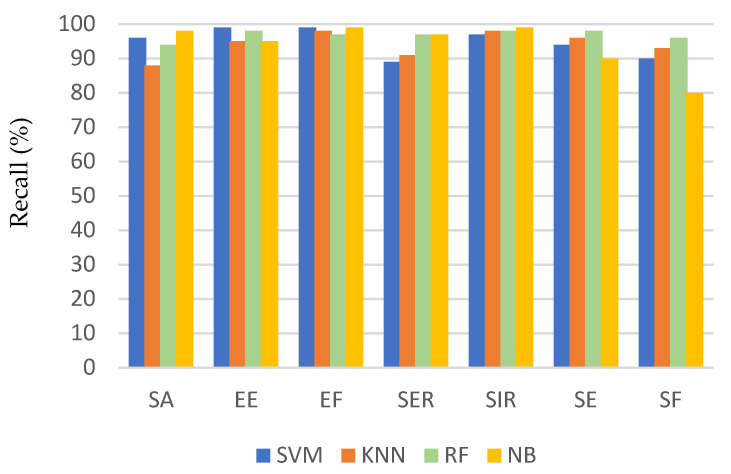
Recall for each activity using different classification algorithms.

**Figure 7:**
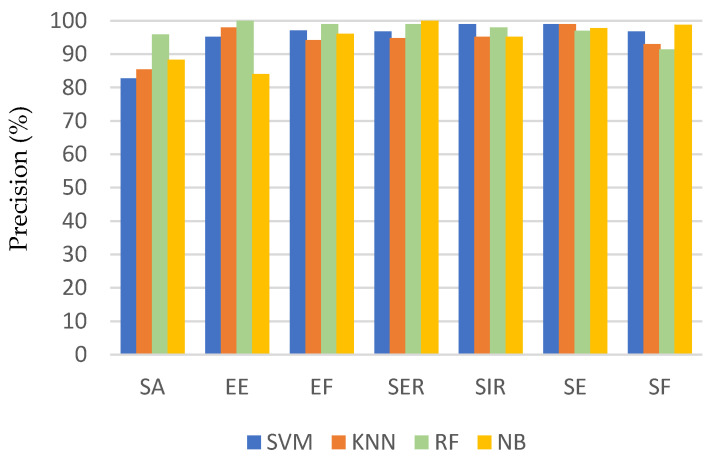
Precision for each activity using different classification algorithms.

**Figure 8:**
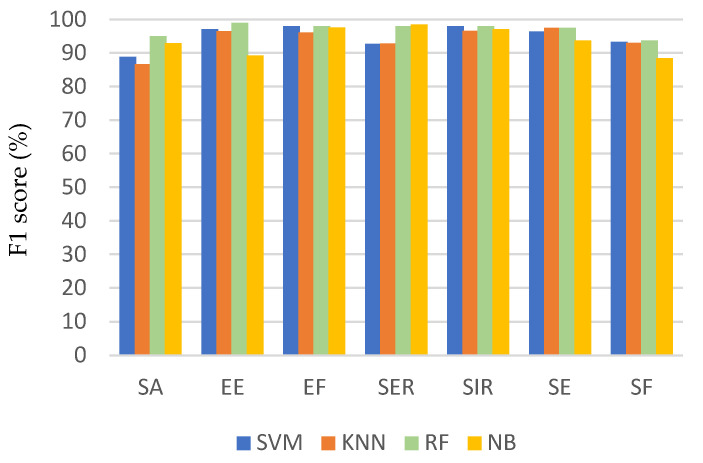
F1 score for each activity using different classification algorithms.

Figure 6, Figure 7 and Figure 8 show a comparison of the performance of the four classifiers on each activity, where the bars in the clusters represent the average recall, precision, and F1 score, respectively. Generally, most of the activities achieved a high recognition rate when classified by all the algorithms. Among the activities, the recall of flexion was the worst, especially for the SVM and NB classifiers, but it was still associated with a recognition accuracy of 80%. This can be attributed to the fact that these two classifiers frequently misclassified SF as SA or EE. Consequently, the precision of recognizing SA and EE was the lowest when using these two classifiers, but they achieved an average precision ranging from 80% to 88%.

Table 3, Table 4, Table 5 and Table 6 show the confusion matrices for each activity class obtained using the SVM, KNN, RF, and NB classifiers, respectively, on the balanced dataset, which consisted of 700 samples. As shown, a vast majority of the samples lays along the diagonal. In all classifiers, the method commonly confused SA and SF. This is to be expected because these activities are regarded as similar, particularly in terms of the starting and ending points of the activity, as well as the range of motion. However, the proposed method with the RF classifier achieved a recognition accuracy of 94% for SA and 96% for SF.

To investigate the effectiveness of the proposed method using the dynamic segmentation technique, the results were compared with the sliding window, which is the most widely used approach. To accomplish this, different window lengths were evaluated, including 2 s, 2.5 s, and 3.5 s, with an overlap of 50%. These values were chosen based on a similar study that obtained the highest recognition accuracy using a 2-s window length [12]. In addition, the results provided by [41] demonstrated that window lengths between 2.5–3.5 s provide the best tradeoff between performance and latency. Table 7 reports the comparison results for each activity using F1 score, as well as the overall accuracy obtained using each technique.

## 4. Discussion

In this study, we proposed and verified a systematic activity recognition method to identify physical activities performed during the rehabilitation of individuals with SCI. The empirical results indicate the effectiveness of the method in recognizing all the activities considered in the study. Furthermore, the results demonstrate the applicability of the method on continuous data collected from a single wearable sensor.

The proposed method achieved a high recognition accuracy of 96.86% when classifying various physical activities. This result clearly shows that data from wearable sensors, when used with classification algorithms, can contribute to a valuable technology for performing automated rehabilitation assessments.

Different machine learning algorithms were used in the proposed method, including SVM, KNN, RF, and NB. As shown in Table 2, RF outperformed the other algorithms with a statistically significant result. This may be attributable to the multiple decision trees involved in RF, where RF combines various discriminative rules to improve the processing of continuous data. Moreover, RF is a classifier that can provide its near-optimal (or even optimal) results without the need for complex parameter tuning [22].

The performance evaluation for each activity shown in Figure 6, Figure 7 and Figure 8 shows that the method encountered the greatest challenges when recognizing similar activities (e.g., SA and SF). However, the recognition rate reached 98% and 96% for SA and SF, respectively, depending on the classifier used. This demonstrates the robustness of the proposed method to differentiate between activities that involve similar movements.

Table 7 reports our results compared to the sliding window, which was the most common segmentation approach applied in similar prior studies [12]. The comparison demonstrates that the proposed method enabled higher classification performance for all the provided activities. The level of improvement was even higher for similar movements such as SA and SF, where the sliding window frequently confused these activities and experienced difficulties in differentiating between them. In short, our method achieved an overall accuracy of 96.86%, which was higher than 91.86%, 91.58%, and 87% for the sliding window with window lengths of 2 s, 2.5 s, and 3.5 s, respectively.

The method presented in this study overcomes the limitation of patients’ self-reported measures and surveys. At present, self-reported measures and surveys are routinely used to verify the completeness of rehabilitation activities applied in SCI therapy. The proposed approach can also be used to evaluate the quality of the activities from two aspects. First, performance correctness, which can be undertaken through activity recognition (i.e., since the developed model will not recognize the activity unless it has been performed correctly). The second aspect is repetition counts, which can also be provided by our approach. On the other hand, other quality aspects such as intensity cannot be evaluated by the proposed approach and should be self-reported. With the regard to adaptability, the method is expected to achieve the same performance. This is because this work focused on the type of SCI, where patients have healthy upper limbs but need rehabilitation to avoid low muscular strength and other associated symptoms, as mentioned earlier. Of note, the method will be introduced into hospital-based rehabilitation sessions to examine the performance on SCI individuals.

Given that the proposed approach has certain limitations, many issues remain open, particularly those relating to the improvement of recognition performance. First, in the current implementation, only time-domain features were used. Consequently, extracting additional features in the frequency domain could potentially improve recognition accuracy. Secondly, an applicable feature selection method can be used to reduce the dimensionality of the problem and enable more efficient implementation of the predictive model. Thirdly, a majority voting approach could be performed over a range of consecutive classifiers for further performance improvement. Finally, additional signal processing techniques will be applied, especially for the attenuating of artifacts, which might be introduced to the signal due to body movements and affect the recognition accuracy of the model.

## 5. Conclusions

This study introduced a novel application of activity recognition to assist in the rehabilitation of SCI patients. An experiment was conducted to verify the proposed method and build the predictive model. The results, which were generated using a single wrist-worn accelerometer, attest to the effectiveness of the proposed approach and its applicability in healthcare self-management (e.g., telerehabilitation programs). In addition, compared to the sliding window, our approach achieved higher performance in recognizing all of the physical activities under study.

## Figures and Tables

**Table 1:** List of features used (notation: a∈(x,y,z,m); *N* is the number of data points; *i* is the index).

Name	Definition
Minimum	lowest *a_i_*, *i* = 1, 2, …, *N*
Maximum	highest *a_i_*, *i* = 1, 2, …, *N*
Range	max(*a*)–min(*a*)
Mean	$\frac{1}{N}\;\sum_{i=1}^{N}a_{i}$
Standard Deviation	$\sqrt{\frac{1}{N}\;\sum_{i=1}^{N}{\left(a_{i}-\bar{a}\right)}^{2}}$
Root Mean Square	$\sqrt{\frac{1}{N}\;\sum_{i=1}^{N}a_{i}^{2}}\;\;$

**Table 2:** Performance evaluation of the proposed method, including accuracy, recall, precision, and F1 measures (mean ± standard deviation).

	Accuracy	Recall	Precision	*F*1 Score
SVM	94.86% ± 5.5%	94.86% ± 4.1%	95.21% ± 5.7%	94.91% ± 3.5%
KNN	94.15% ± 3.6%	94.15% ± 3.8%	94.22% ± 4.5%	94.16% ± 3.8%
RF	96.86% ± 4%	96.86% ± 1.5%	97.2% ± 2.9%	97.02% ± 2%
NB	94% ± 6.1%	94% ± 7%	94.33% ± 6%	93.91% ± 4.1%

**Table 3:** Confusion matrix for SVM classifier (in %). Cells with value 0 are left blank.

Actual Activity	Predicted Activity						
	SA	EE	EF	SER	SIR	SE	SF
SA	96	1					3
EE		99			1		
EF			99			1	
SER	9	2		89			
SIR	1	2			97		
SE			3	3		94	
SF	10						90

**Table 4:** Confusion matrix for KNN classifier (in %). Cells with value 0 are left blank.

Actual Activity	Predicted Activity						
	SA	EE	EF	SER	SIR	SE	SF
SA	88	2	2	5			3
EE		95	4		1		
EF			98				2
SER	8			91		1	
SIR					98		2
SE						96	
SF	7						93

**Table 5:** Confusion matrix for RF classifier (in %). Cells with value 0 are left blank.

Actual Activity	Predicted Activity						
	SA	EE	EF	SER	SIR	SE	SF
SA	94						6
EE		98			2		
EF			97				3
SER				97		3	
SIR			1	1	98		
SE				2		98	
SF	4						96

**Table 6:** Confusion matrix for NB classifier (in %). Cells with value 0 are left blank.

Actual Activity	Predicted Activity						
	SA	EE	EF	SER	SIR	SE	SF
SA	98					1	1
EE		95	4		1		
EF			99			1	
SER	1	2		97			
SIR		1			99		
SE		6			4	90	
SF	12	8					80

**Table 7:** Performance comparison of different segmentation techniques using F1 score and overall accuracy.

	SA	EE	EF	SER	SIR	SE	SF	Overall Accuracy
Sliding window (2 s)	87.44%	95.48%	94.48%	93.47%	94.48%	93%	86.14%	91.86%
Sliding window (2.5 s)	88.56%	94.42%	90.65%	91.93%	93.88%	93.54%	87.81%	91.58%
Sliding window (3.5 s)	82.42%	90.46%	88.56%	88.45%	89.56%	88.56%	81%	87%
Our method	94.96%	98.99%	97.98%	97.98%	98%	97.52%	93.66%	96.86%
